# Review of serum prolactin levels as an antipsychotic-response biomarker

**DOI:** 10.15406/oajtmr.2018.02.00043

**Published:** 2018-05-04

**Authors:** Judith M Gault, Abraham M Nussbaum

**Affiliations:** 1 Departments of Psychiatry, University of Colorado Denver, Anschutz Medical Campus, USA; 2 Departments of Neurosurgery, University of Colorado Denver, USA; 3 Denver Health, USA

**Keywords:** antipsychotic, dopamine, biomarker, prolactin, dose response, schizophrenia, psychosis, serum

## Abstract

Antipsychotics acting as antagonists at dopamine D2 receptors concentrated in the striatum are the cornerstone of effective treatment of psychosis. Substantial progress in treating persons with schizophrenia could be achieved by the identification of biomarkers which reliably determine the lowest efficacious dose of antipsychotics. Prolactin levels have been considered a promising treatment-response biomarker due to dopamine’s regulation of serum prolactin levels through D2 receptors in the hypothalamic-pituitary pathway. Prolactin secretion in response antipsychotic administration is associated with the antipsychotics affinity for D2 receptors. This review assesses the available literature on the use of serum prolactin levels as an antipsychotic-response biomarker. Articles were identified through PubMed as well as the reference lists of full text articles available online. Relevant publications were summarized briefly to define the limitations and utility of serum prolactin levels as a tool for improving antipsychotic dosing. Serum prolactin levels in combination with prolactin-inducing potencies for each antipsychotic may help identify the lowest effective dose of antipsychotic medications. , In addition to the fact that prolactin secretion is dependent on serum antipsychotic levels and not brain levels, recent findings show that prolactin release is independent of the β-arrestin-2 pathway and GSK3β regulation, one branch of the pathway that has been implicated in antipsychotic efficacy. Therefore, serum prolactin is an indirect biomarker for treatment response. Further investigations are warranted to characterize prolactin-antipsychotic dose-response curves and systematically test the utility of measuring prolactin levels in patients to identify a person’s lowest efficacious dose.

## Introduction

The current standard of care for optimizing antipsychotic dose relies on titration within a recommended dose range while subjectively assessing how symptoms respond to dose changes.^1^ Using this general approach to dose finding, antipsychotics are clearly efficacious and are fundamental to treatment success for persons with schizophrenia. Antipsychotic clinical response is high; for example, 94% of first-episode patients who are hospitalized with SZ clinically respond to olanzapine or risperidone.^2,3^

Over 90% of subjects in clinical trials experience significant (50-70%) to minimal improvement (20-30%) on antipsychotics and 10-20% show no improvement or decline without a significant difference between first- and secondgeneration antipsychotics, with the exception of clozapine.^4-6^ Antagonistic action at dopamine D2 receptors, found at their highest concentration in the striatum, is essential to antipsychotic action (Figure 1A). Nevertheless, currently available treatments have several notable limitations. Antipsychotics have similar efficacy with the exception of the most effective antipsychotic, clozapine.^7-9^ A multicenter clinical trial showed that 74% of the patients enrolled discontinued their medication because of poor efficacy and intolerable adverse effects.^6^ Even when compliant with medications, patients relapse at a rate of 27-35% per year even when compliant with medication.^1,10^ Antipsychotics provide symptomatic relief, but are not curative, and are sometimes inadequate even when an optimal dose is administered.

Therefore, patients may be subjected to excessive doses that increase adverse effects without additional therapeutic efficacy. It is established that a specific antipsychotic dose will result in variable patient response and 45-fold differences in drug levels in the plasma.^11^ Most antipsychotic are readily absorbed through the gastrointestinal tract, are metabolized by various cytochrome P450 enzymes primarily in the liver, and are highly bound by plasma proteins.^12^ Antipsychotics are lipophilic and readily cross the blood brain barrier (BBB) to concentrate possibly 10-20 times higher in the brain. While measuring plasma levels of the mood stabilizer, lithium, has been successful in maximizing efficacy and minimizing adverse effects, monitoring blood levels of antipsychotics has been less successful and conflicting studies have made it difficult to establish guidelines for the efficacious range of antipsychotic therapeutic serum concentrations.^13,14^ Several studies have shown that a 64-80% response rate is achieved after 6 months at plasma levels of clozapine of greater than 350-450 ng/mL.^11,15-17^ For other antipsychotics, the range of clinically efficacious plasma levels is not agreed upon and study results areinconsistent or requires further confirmation. At present, plasma levels are helpful as proxies for medication adherence, demonstrating that a reasonable level is found in nonresponders, and avoiding toxicity, but have not been useful for optimizing an efficacious dose.

While further investigation into efficacious serum concentrations for antipsychotics is conducted, additional objective measures are needed to identify the lowest efficacious dose while minimizing adverse effects. This paper assesses the potential use of serum prolactin levels to determine the optimal dose of some antipsychotic medications and the lessons learned from measuring prolactin levels with antipsychotic treatment.

## Discussion

### Prolactin biology

Discovered in humans in the early 1970’s^18^ prolactin is a 23 kDa polypeptide hormone predominantly synthesized and secreted by the lactotroph cells of the anterior pituitary into the third ventricle.^19^ The release of prolactin from the pituitary is regulated by the hypothalamus and a complex array of feedback mechanisms. The release of prolactin is stimulated by substances like oxytocin and thyrotropin releasing factor, but inhibited by dopamine, somatostatin, and γ-aminobutyric acid (GABA) and prolactin itself. The pituitary contributes primarily to prolactin levels and primates and rats with their pituitary removed have an 80-95.4% reduction in serum prolactin.^20,21^ Dopamine is the main factor inhibiting prolactin release. Dopamine receptors on the cell membranes of lactotrophs are primarily D2 dopamine receptors (Figure 1B). Dopamine neurons project from the dorsomedialportion of the hypothalamus’s arcuate nucleus to the external zone of the anterior pituitary’s median eminence along the tuberoinfundibular pathway, delivering dopamine through the portal circulation into the sinusoid capillaries of the anterior lobe.^22^ When the release of dopamine is disrupted or its activity on the anterior pituitary is blocked, prolactin is released into the systemic circulation. Serum prolactin studies are widely available at clinical laboratories. The prolactin assay is performed on serum, in a serum separator tube within forty minutes of the blood draw. The test costs approximately fifteen dollars and normal levels range from 1–25 μg/L for women and 1–20 μg/L for men.^23^

The release of dopamine from the tuberoinfundibular pathway is inhibited by cholecystokinin-8, endogenous opioid peptides, estrogen, galanin, histamine, nitrous oxide, norepinephrine, serotonin, somatostatin, and γ-aminobutyric acid. The release of dopamine from the tuberoinfundibular pathway is stimulated by acetycholine, angiotensin II, atrial natriuretic peptides, bombesin-like peptides, calcitonin, neuropeptide Y, glutamate, neurotensin, oxytocin, thyrotropin releasing factor, vasoactive intestinal polypeptide, and vasopression.^22^ The clinical implication is that because the release of prolactin is influenced by so many neurotransmitters and hormones, serum prolactin can be elevated by multiple drugs often used by people with mental illness including agents that increase serotonin, including tricyclic antidepressants and selective serotonin reuptake inhibitors, drugs that antagonize histamine receptors, like cimetidine and ranitidine, hormones in oral contraceptives, and drugs of abuse, including alcohol and opiates.^24^

### Antipsychotic induced hyperprolactinemia

Antipsychotics bind to the short (presynaptic autoreceptor), long (post synaptic) and the longer (less abundant) forms of D2 receptors^25,26^ to lower dopaminergic tone in the meso-cortical and meso-limbic pathways and treat the psychotic symptoms of SZ.^27-31^ Although D2 receptors have a high-affinity and low–affinity state, most D2 receptors are in the high affinity state, the state where antipsychotics bind.^32^ Dopamine acting on D2 receptors is inhibitory and D2 receptors are at highest levels (16.5 pmol/g tissue) primarily on medium spiny neurons in the striatum.

Many adverse effects of antipsychotic use are due to antagonism at D2 receptors in the nigro-striatal pathway (parkinsonian, extrapyramidal side effects) and the tubero-infundibular (prolactin) pathway.^33,34^ Regions of the tubero-infundibular pathway have D2 receptors levels are 1.3 to 1.8 pmol/g tissue in the pituitary and hypothalamus respectively.^35^ Prolactin released by other regions of the brain, lymphocytes and other organs is thought to contribute up to 20% total prolactin levels than the anterior pituitary and it is currently not known whether these secondary sites are also altered by high affinity D2 antagonism. Hyperprolactinemia is defined by prolactin levels above 15-18.77 ng/ml for males and 23-24.20 ng/ml in females.^36-38^

There is conflicting evidence that prolactin levels maybe elevated during acute psychosis. Prolactin levels were elevated 2.3-fold (15.1 and 35.1 ng/ml in men and women respectively) in 33 antipsychotic-naïve subjects with SZ (n=18), brief psychotic disorder (n=9), schizophreniform disorder (n=3) and unspecified psychosis (n=3).^39^ However, a 2.1-fold decrease in serumprolactin levels was found in 30 male hospitalized patients with SZ at baseline possibly in response to antipsychotic withdrawal during the 2-week wash out period.^40^ In another study prolactin levels were found at the high end of normal at baseline (18.2 and 23.5 ng/ml for men and women respectively) in 27 acutely psychotic patients with SZ when they were medication-naïve or medication-free for at least 4 weeks.^41^ One might expect subjects experiencing psychosis to have low prolactin levels if psychosis is related to excessive dopamine stimulation at the D2 receptors or because psychosis causes a lack of sleep. Increased prolactin levels have been implicated as one of 51 SZ diagnostic biomarker for treatment naive subjects.^42^ In 14 postmortem brains from SZ subjects, prolactin levels were reduced by 46% relative to controls although only one of the two assessments were found significant.^43^ These findings warrant further investigation to determine whether prolactin levels are elevated in relation to physical/emotional stress during psychosis or if they are generally elevated in treatment-naïve subjects with SZ. The prevalence of hyperprolactinemia when taking prolactinraising antipsychotics is 65.6% in women of reproductive age, 45.1% in postmenopausal women and 42.4% in men.^38^

Hyperprolactinemia is potentially associated with amenorrhoea, galactorrhoea, sexual dysfunction,^44^ breast engorgement, and osteoporosis.^37,45^ Neuroscientists began investigations into prolactin levels as an indicator of hypothalamic activity during psychiatric illness^19^ and following the observation in animals that many antipsychotics stimulate prolactin secretion^46,47^ consistent with prolactin levels being an indicator of dopamine blockade and generating the hypothesis that increased serum prolactin levels were associated with antipsychotic clinical efficacy. However, prolactin levels are inconsistently correlated to efficacy; for example clozapine is the most efficacious antipsychotic, but it does not cause a sustained increase in prolactin levels (Table 1 ).^48,49^ Prolactin levels cannot be used to predict who will be responsive to antipsychotic drugs even though they might be helpful in determining if a dose is too low. The potential use of prolactin as a measure of neuroleptic bioavailability and central nervous system activity was the focus of a review article in 1983.^50^ Brown summarized compelling evidence that showed an association between prolactin levels with antipsychotic dose in 26 men with SZ being titrated off neuroleptics, “fairly consistent” findings of correlations of 0.83/0.92 between prolactin and haloperidol/chlorpromazine levels throughout the therapeutic range, studies showing low serum prolactin levels in patients that had relapsed, a 20-25% variability in an individual’s prolactin levels when they are a constant dose and that “serum prolactin is far more affected by dose, serum level and individual differences than by physiological adaptive changes of tolerance”.^50^ In a preliminary study done in patients with SZ, genetic variation at the prolactin and prolactin receptor loci were not associated with: 1) prolactin serum levels;^51^ 2) patients who do not respond to antipsychotic treatment; or 3) patients who have tardive dyskinesia.^52^ Serum prolactin levels are primarily under dopaminergic control and increase in response to D2 antagonists such as haloperidol, blockade of monoamine transporters that decrease extracellular dopamine such as reserpine, but do not change significantly in response to partial D2 agonists such as aripiprazole.^53^

### Antipsychotic-specific induction of prolactin levels

**Figure 2:**
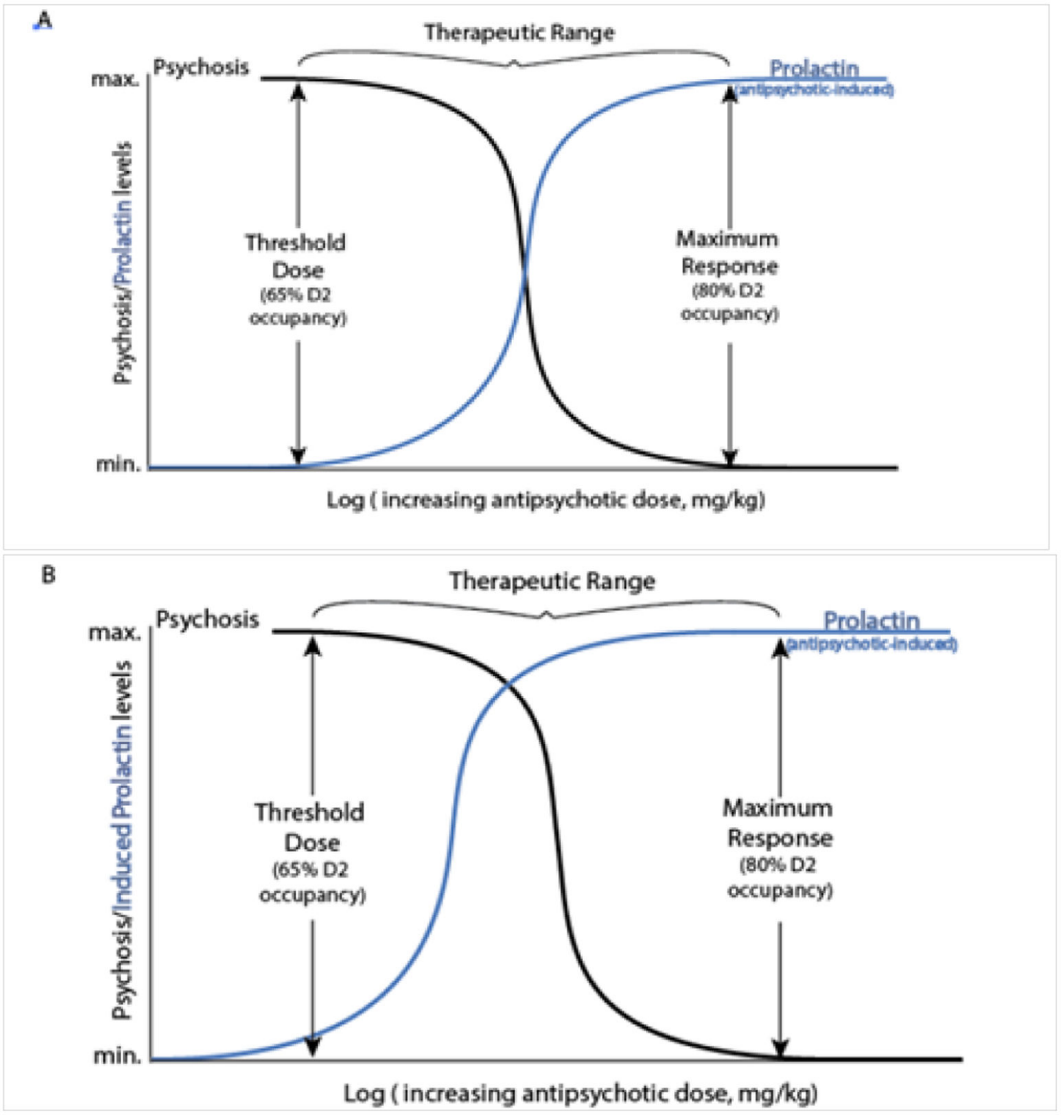
**(A)** Hypothetical dose-response curves. Depiction dose-response curves with similar threshold doses where antipsychotic effect occurs along with antipsychotic-induced prolactin secretion in the plasma. (B) Depiction of dose-response curves where the threshold of antipsychotic-induced prolactin secretion occurs before the threshold dose of antipsychotic effect.

Compelling evidence supporting a correlation (r2=0.52) between the prolactin inducing potency (Table 1) and lowest effective antipsychotic dose using 19 antipsychotics was identified by extrapolating to a haloperidol dose-prolactin increase response curve.^54^ The potential use of antipsychotic-induced serum prolactin levels as a biomarker of the lowest effective dose of antipsychotic was identified in 95% of the studies examined presented.^54^ De Visser et al. recommended generating doseprolactin response curves specific to each antipsychotic. They did not discuss whether prolactin levels could be helpful for identifying an individual’s lowest effective dose as proposed here (Figure 2 A) & (Figure 2 B).^54^ Relying on studies reporting Risperidone, amisulpride and paliperidone have the greatest risk (80-90% of females) for hyperprolactinemia followed by first generation medications (75% risk) then other second-generation antipsychotics have lower and less sustained effects olanzapine (78% risk) then ziprasidone (73% risk) then quetiapine (59% risk) and clozapine.^45,55^ Aripiprazole, a partial D2 agonist has no effect on prolactin levels and can be used to reduce prolactin levels.^56,57^

Peripheral prolactin levels were associated with peripheral levels of some antipsychotic medications.^58,59^ Prolactin levels were associated with D2 occupancy as determined by PET scan after taking haloperidol^60^ and positively associated with D2 striatal occupancy in humans. In rats, D2 receptor occupancy of the striatum and pituitary was correlated 1 hr after administering 0.01-2.5 mg/kg doses of olanzapine, 0.04-40 mg/kg of risperidone, and 0.16-40 mg/kg of quetiapine.

### Domperidone and amisulpride

The lactotroph cells of the anterior pituitary are accessible and respond with prolactin release to D2 antagonists acting peripherally including domperidone that does not readily cross the blood-brain barrier (BBB).^61-63^ Despite the fact that the pituitary is vascularized by peripheral blood, antipsychotics may be at comparable concentrations to other brain regions because antipsychotics readily cross the BBB with the exception of amisulpride,^64^ brain regions are highly vascular may have similar propensities to bind lipophilic drugs, and many regions of the brain are secondary sites for prolactin synthesis and may also be under dopamine control. For amisulpride, prolactin levels are not correlated with central D2/D3 occupancy or plasma levels of drug possibly because pituitary D2/D3 occupancy is saturated while central D2/D3 levels are not.^65,66^

The fact that amisulpride plasma levels were associated with D2/D3 occupancy in the striatum, thalamus and temporal cortex (r=0.83-0.87, p<0.05) is consistent with the theory that peripheral biomarkers may be useful even for drugs like amisulpride with restricted BBB permeation as long as scale of assessment is linear throughout the dose range and does not plateau due to the high D2 occupancy in the periphery.^66^

### Risperidone

Risperidone causes a rapid and persistent elevation of prolactin levels. Unlike amisulpride, which has a striatal to pituitary potency ratio of 654, risperidone’s ratio of 14 is closer to quetiapine’s ratio of 6 in rats.^65^ Serum levels of risperidone’s primary metabolite, 9-OH-risperidone, is significantly correlated with prolactin levels (r=0.52, n=25, p=0.008) while levels ofrisperidone were not (r=0.23, p=0.26).^67^ Although both risperidone and 9-OH-risperidone, which is commercially available as paliperidone, have similar affinities to D2 receptors, 9-OH-risperidone has a lower brain/plasma ratio and a half-life of 20 hours in contrast to risperidone’s 2-4 hrs half-life. Prolactin levels were weakly correlated with ~4-6 mg/day daily dose of risperidone (r=0.328, p=0.001) and showed a trend towards being weakly associated with improvement of in psychosis assessed at baseline without treatment for 2-4 weeks and then again at 12 weeks of risperidone treatment (r=−0.329-0.51, p=0.016-0.094).^40,41^ Prolactin levels peaked within 2-4 weeks while BPRS scores continued to improve over the entire 12 weeks.^41^

### Haloperidol

Early use of high doses of haloperidol and saturating prolactin response has been invoked as contributing to the conflicting reports concerning prolactin level correlation with clinical response^68^ and Chou et al. found prolactin levels were correlated with positive symptom improvement in 4 women and 19 men experiencing acute psychosis upon hospitalization who were medication free for at least 6 days then treated with 15 mg/day resulting in plasma levels of 6.6+/−4.7 ng/ml (r=−0.54, p=0.008).^68^ An earlier study with 0.25 mg/ kg/day haloperidol found a correlation between the ratio of prolactin to homovanillic acid, a principal dopamine metabolite, and patient improvement in positive symptoms in 22 men and 16 women after only 4 days of treatment; participants were diagnosed with either SZ or schizophreniform disorder and were medication free 4 months at baseline (r=−0.52 in women and p=−0.42 in both men and women combined, p=0.006).^69^

### Olanzapine

D2 occupancy in the pituitary is closely correlated with D2 occupancy in the striatum in rats over the 60-96.5% occupancy range shown to be efficacious in humans (Table 1). Decreases in prolactin levels were associated with improvement of positive symptoms (p=0.002) in 36 men and 24 women with the diagnosis of SZ who had stopped taking antipsychotics at least 7 days before starting 3 months of treatment with olanzapine at 10-30 mg/day.^70^ Subjects were previously taking olanzapine,^27^ first-generation antipsychotic,^18^ risperidone,^12^ clozapine,^2^ or quetiapine.^1^ Serum prolactin levels were 41.63+/−23.63ng/ml at baseline and reached their lowest levels in the second month at 25.72+/−16.77 ng/ml.

### Timing of prolactin assessment

Serum prolactin levels were monitored within hours of administering antipsychotic medication in the de Visser et al study that identified a significant effect with antipsychotics doses (Table 1 ).^54,71,72^ Ideally biomarker assessment should occur when levels of drug reach steady-state concentrations and levels centrally are correlated with peripheral levels around 4-5 half-lives after a change in dose as is done with blood levels of lithium.^1^ It should be determined if antipsychotic levels in early treatment are correlated with steady-state levels. Plasma levels of both haloperidol and the active haloperidol metabolite added together are correlated with D2 occupancy (r2=0.84) at 12-14 hrs after their last dose maintained over a week.^73^

Unlike most second-generation antipsychotics with a half-life of <3 days that reach a steady state within 2 weeks, the half-life of haloperidol is 3 weeks and reaches steady-state concentrations after 100 days.^1^ Prolactin levels rise within 30 minutes of administering haloperidol, beginning to level off in males at 60 minutes and >90-120 minutes in females. Peak prolactin levels are reached around 10 days and remain elevated from baseline even 20 weeks from administering haloperidol or risperidone.^37^ Prolactin levels peak then decline to a stable level after 4 months that is maintained for years of extended antipsychotic use.^74^

Peripheral prolactin levels are associated with D2 occupancy of some antipsychotic medications.^58,59^ Prolactin levels are correlated with D2 occupancy after 50% occupancy is reached.^75^ For example, while determining the transient D2 occupancy tested at 1, 2.5 and 20 hours after an initial dose of quetiapine, D2 occupancy levels were noted to peak at 2.5 hrs(62%) and were correlated with prolactin levels (r2=0.8) and plasma levels (r2=0.96); after 4 weeks when levels of quetiapine peaked prolactin levels were elevated, but at 12 weeks of treatment prolactin levels were normal.^59^ While investigating the mechanism of increased prolactin response to 2-20 mg/day of haloperidol, prolactin levels were found correlated with D2 occupancy (r=0.45-0.5).^58^ However, a study of rats receiving risperidone and amisulpride found that D2 occupancy in the pituitary is higher than in the striatum. Although antipsychotics readily cross the BBB, functional genetic variation and environmental influences in BBB efflux (P-glycoprotein) or transporters may contribute to interindividual differences in the proportion of antipsychotic centrally relative to peripheral levels.^76^

Finally, to be an effective biomarker of treatment response, the range of effective prolactin levels would have to be determined for each drug because of differences in D2 and 5-HT receptor affinity. Prolactin levels have been correlated with serotonin levels in 25 healthy controls (r=0.48, P<0.05)^77^ and levels appear to be partially regulated by serotonin as shown by 5-HT antagonists decrease PRL levels.^78^ Alternately, individual dose-response curves could be generated while the patient’s dose is titrated in order to identify the point of therapeutic efficacy.

## Conclusions

There are several limitations in using prolactin levels as a biomarker of antipsychotic-response including:

- The anterior pituitary is a circumventricular organ and prolactin is released in response to free antipsychotic in the peripheral blood as shown by the D2 antagonist, domperidone, that does not readily cross the blood brain barrier.^63^
- Serum prolactin levels are influenced by several neurotransmitters in addition to dopamine.
- Hyperprolactinemia is induced by antipsychotics with moderate to high affinity for D2 receptors; 4) it is not known whether measuring prolactin levels would be better than measuring the free levels of antipsychotics in the serum; and prolactin may be induced at low doses of antipsychotics.^72^

There may be a number of potential advantages to using a biomarker like prolactin that is released in response to D2 receptor antagonist and DA agonist occupancy. While smoking, absorption and genetic factors of cytochrome P450 enzymes that contribute to individual differences in the breakdown of antipsychotics in the liver^79,80^ would be accounted for by measuring either plasma levels or prolactin levels, measuring prolactin levels takes into account individual conditions that may change the free fraction of drug circulating,^1^ an individual’s genetic and environmental factors that contribute to the numbers of D2 receptors available such as upregulation of receptors in response to antipsychotic drugs. In addition, measuring prolactin levels is inexpensive, may be useful for determining the minimal dose of antipsychotics, and a single test would take into account all the active metabolites. Multiple tests throughout dose titrations might allow generation of a person’s specific dose response which might allow prediction of a personal lowest efficacious dose of a specific antipsychotic medication instead of relying solely on data generated for a population. There is inter-individual variability in D2 receptor density that appears to be affected in part by age^81,82^ and having a biomarker that is in a regulatory feedback loop may account for changes in D2 receptor density and occupancy.^83-95^

## Figures and Tables

**Figure 1:**
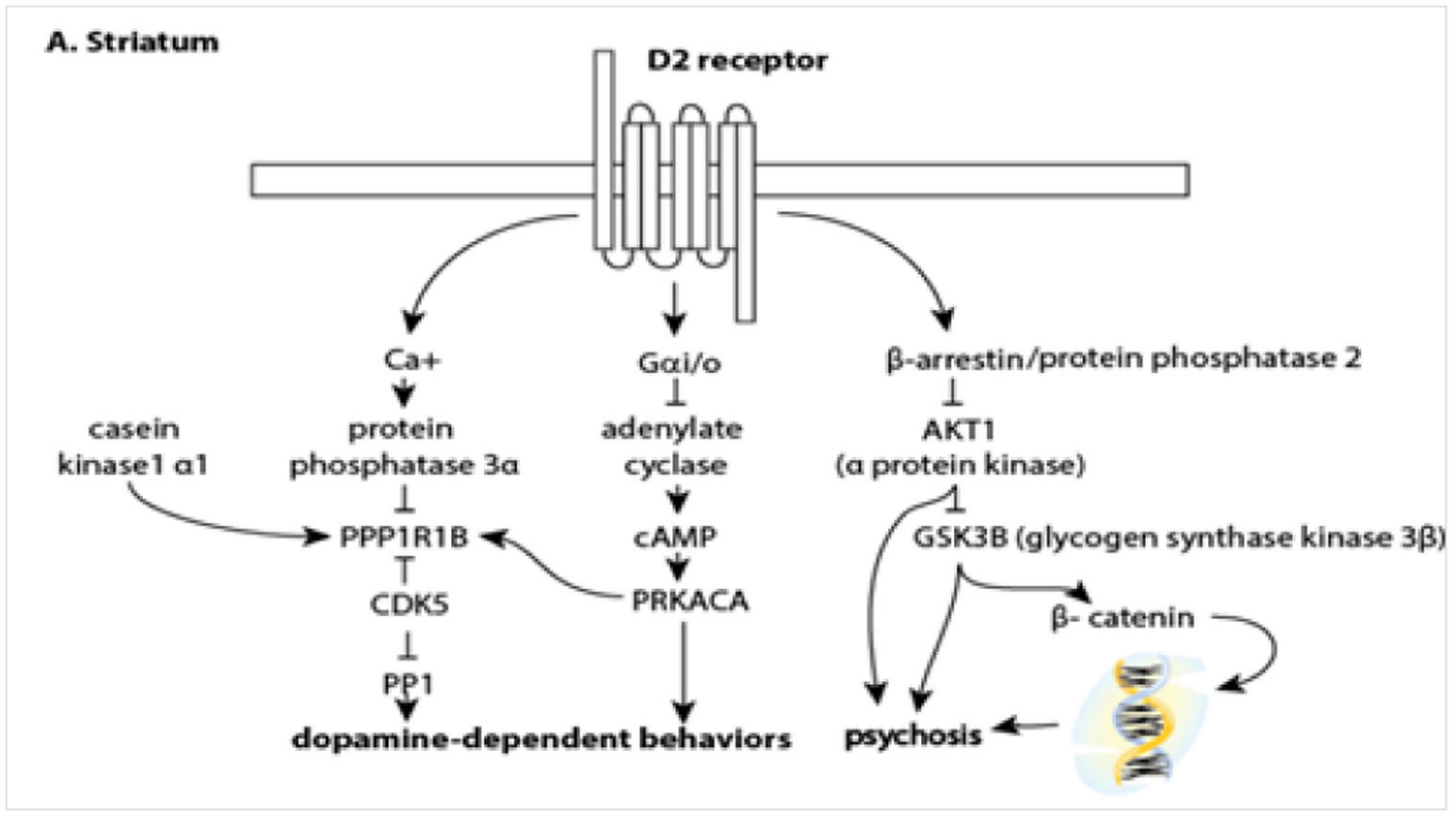
**(A)** The D2 receptor pathway found in the striatum. The β-arrestin pathway has been shown to contribute to antipsychotic effect.

**Figure 1:**
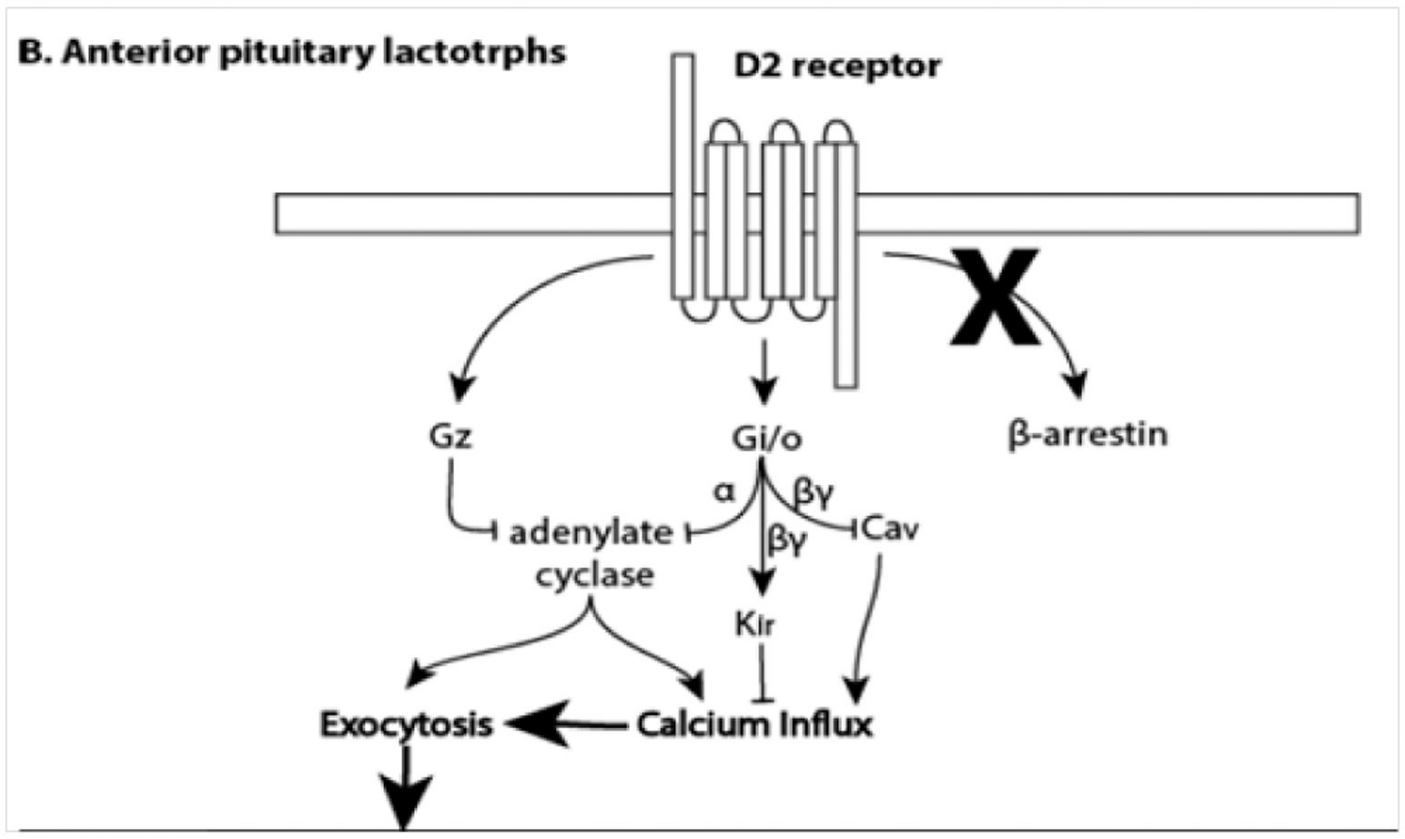
**(B)** The D2 pathway found in the anterior pituitary. Prolactin secretion occurs through dopamine D2 receptors through G(i/o) proteins that block voltage-gated Ca(2+) influx and G(z) signaling by desensitizing Ca(^2+^) secretion. The D2/β-arrestin-2 pathway involving GSK3β does not appear to be involved in prolactin release.93–95 AKT1, alpha protein kinase serine/threonine kinase 1, CDK5, cyclin dependent kinase 5; GSK3β, glycogen synthase kinase 3 beta; PP1, protein phosphatase 1 catalytic subunit alpha; PPP1R1B, protein phosphatase 1 regulatory inhibitor subunit 1B; PRKACA, protein kinase cAMP-activated catalytic subunit alpha.

**Table 1:** Summary of select antipsychotic drugs on prolactin levels

Drug	Dose range mg/day	Protein binding	Active metabolite	Half-life hrs/steady state days	Prolactin-inducing dose equivalents(54)/induces hyperprolactinemia	Striatum D2 occupancy (mg/day)striatum
Amisulpride	300-1200				56.6/Yes	85%(83)
Aripiprazole (Partial Agonist)	10-30	98%	Dehydo-ARIP	75-146/14	−/No (53)*	50-94% (5-50-94% (5-30) (83,84)
Chlorpromazine	30-300	>90%	none	30	39.6/−	
Clozapine	300-900	97%	norclozapine	12/3-4	309/No (49,85)	30-70%(100-800)(83,86,87)
Fluphenazine	2.5-40	N/A	N/A	N/A	1.89/Yes	
Haloperidol	1-15	92%	none	3 weeks/ 100	1.11/Yes	60-91.9% (2-5)(73,83)
Loxapine	60-250	N/A	none	4	−/Yes(72)	60-80%(15-30)(88)
Olanzapine	5-20	93%	none	20-70/7	−/No (49)	60-96.5% (5-40)(83,86,89)
Quetiapine	300-400	80%	none	1.5/2-3	−/No (49)	49.1-64%(150-600)(83,90)
Risperidone	2-8	89%	paliperidone	20	0.1/Yes(49)	65->92.4% (2-6)(83,86,91)
Ziprasidone	40-160	>99%	none	5-10/1-3	−/No(92)	82.9%(83)

* Fold change over baseline vs after medication in healthy controls, M: male and F: female

Association of prolactin levels with antipsychotic dose.

Timing and duration of prolactin response to antipsychotic dose.

## Abbreviations:

AKT1: alpha protein kinase serine/threonine kinase 1
BBB: blood brain barrier
BPRS: brief psychiatric rating scale
CDK5: cyclin dependent kinase 5
D2: dopamine D2 receptor
GSK3β: glycogen synthase kinase 3 beta
PRKACA: protein kinase camp-activated catalytic subunit alpha
PP1: protein phosphatase 1 catalytic subunit alpha
PPP1R1B: protein phosphatase 1 regulatory inhibitor subunit 1b
SZ: schizophrenia
GABA: γ-aminobutyric acid
